# Equivalent squares of circular electron fields

**DOI:** 10.1002/acm2.70264

**Published:** 2025-09-24

**Authors:** Patrick N. McDermott

**Affiliations:** ^1^ Department of Radiation Oncology William Beaumont University Hospital/Corewell Health Royal Oak Michigan USA

**Keywords:** electron beams, equivalent squares

## Abstract

**Background/Purpose:**

It is useful to be able to make manual estimates of the output (dose rate) of shaped electron fields. Such estimates can be used to check the treatment planning computed output. For rectangular (or approximately rectangular) fields, the square root rule may be used. Many electron apertures, however, are approximately circular and therefore a method for finding the equivalent square from the radius of circular apertures would be useful.

**Methods:**

A grid of Monte Carlo calculated output values for a variety of square and circular field sizes, applicators, and beam energies of 6 and 15 MeV has been used to find an expression for the equivalent square of circular fields. This equivalence has been tested using a larger data set. The test data set consists of electron energies of 6, 9, 12, and 15 MeV and radii ranging from 1.1 to 5.5 cm, for applicators of size 6 × 6, 10 × 10, 14 × 14, 20 × 20, and 25 × 25 cm^2^. A total of 104 combinations of these parameters have been tested. Some clinical examples are provided to demonstrate how the equivalent square may be used to check treatment planning system MU calculations. Comparisons are made with measured output.

**Results/Conclusions:**

The results show that there is an exceptionally simple and remarkably accurate relationship for the equivalent square of circular electron fields, namely: *X* = 1.83 *R*, where *R* is the radius and *X* is the side length of the equivalent square. This is approximately midway between the two limiting values predicted by Fermi–Eyges theory ($\sqrt{\pi }\leq X/R\leq 2$).

For the 104 combinations of the parameters described above, the average ratio of the circular field output to the equivalent square output is 1.000, and the standard deviation is 0.003. In every case, the accuracy is better than 1% and, in most cases, better than 0.5%. Almost all the ratios fall within the ±0.4% accuracy expected based on the statistical uncertainty in the Monte Carlo calculations. It is shown that the equivalent square rule for circles is more accurate than the square root rule for a range of common widths, *W*, and *L*/*W*, where *L* is the length of the rectangle. For the clinical examples cited, the agreement between estimated and measured output is within a few percent.

## 1 INTRODUCTION

It is useful to have the capability of quickly checking the treatment planning system monitor units for shaped aperture clinical treatment fields. For photon beams, this is done by estimating the equivalent square using the area‐over‐perimeter rule. There is an extensive literature on shaped or irregular electron fields.^1^, ^2^, ^3^, ^4^, ^5^, ^6^, ^7^, ^8^, ^9^, ^10^ Some of these references deal explicitly with equivalent fields. Electron field shaping is accomplished with the use of shaped blocks referred to as “cutouts” that are inserted in an applicator. There have been many efforts over the years to improve techniques for predicting the output for small electron fields in which there is a departure from lateral electron equilibrium. The square root rule has been used for many years to estimate the dose rate for rectangular fields.^11^, ^12^, ^13^, ^14^ Many electron apertures, however, are not even approximately rectangular in nature. A review of patient treatment records in our clinic shows that electron beam apertures commonly used to treat skin cancer and keloid formation are often circular or approximately circular in shape. This is not true of electron breast boost fields, as these fields are generally highly elongated. An exceptionally simple and remarkably accurate expression for the equivalent squares of circular electron fields is introduced here.

The output factor is defined here as:

(1) $$S_{e}\left(f_{a},f_{c},\mathrm{SSD}=100\right)=\frac{{\dot{D}}_{d_{m}}\left(f_{a},f_{c},100\right)}{{\dot{D}}_{d_{m}}\left(10,10,100\right)},$$

where *f_a_* is the applicator size, *f_c_* is the equivalent size of the shaped field, and ${\dot{D}}_{d_{m}}$ is the dose rate (per monitor unit) at the nominal depth of maximum dose (*d_m_*) for the energy of the electron beam. The nominal depth of maximum dose is the depth of maximum dose for a field size of 10 cm by 10 cm. The field size of the applicator and aperture (electron cutout) can be defined at SSD = 100 cm or SSD = 95 cm (Elekta default). In this work, all field sizes are defined at SSD = 95 cm.

For apertures that are rectangular or approximately rectangular, the square root rule is commonly used. The square root rule states that the output for a field of length *L* and width *W* can be computed from a table of output factors for square fields as follows:

(2) $$\begin{array}{cc} & S_{e}\left(f_{a},L\times W,\mathrm{SSD}\right)\\ & \;=\sqrt{S_{e}\left(f_{a},L\times L,\mathrm{SSD}\right)\times S_{e}\left(f_{a},W\times W,\mathrm{SSD}\right)}. \end{array}$$

This rule can be derived from Fermi–Eyges theory.^15^ Many electron beam apertures are circular, or approximately circular, however, and it would be useful to have a rule for dealing with such cases. A simple formula is developed here for the equivalent square of circular fields. The validity of this formula is substantiated by Monte Carlo dose calculations, and it is shown to be accurate to better than 1% for a wide range of energies, applicators, and field sizes. Four clinical examples are given illustrating the application of this equivalence.

## 2 METHODS

From Fermi–Eyges theory, for a rectangular field of length *L* and width *W* at depth *z*, the dose is:

(3) $$\begin{array}{cc} & D\left(x,y,z\right)=\frac{S_{\rho }\left(z\right)\Phi_{T}\left(0\right)}{4\pi A_{2}\left(z\right)}\int_{-W/2}^{W/2}\int_{-L/2}^{L/2}\\ & \;\exp\left\{-\frac{1}{4A_{2}\left(z\right)}\left[{\left(x-x^{\prime }\right)}^{2}+{\left(y-y^{\prime }\right)}^{2}\right]\right\}dx^{\prime }dy^{\prime }, \end{array}$$

*x* and *y* are the coordinates in a lateral plane (perpendicular to the central axis), and the *z* coordinate represents the depth, *S_ρ_* is the mass stopping power, Φ_T_(0) is the incident fluence (assumed uniform across the field), and *A*_2_ is a depth and energy dependent parameter:

(4) $$A_{2}\left(z\right)=\frac{1}{4}\int_{0}^{z}{\left(z-z^{\prime }\right)}^{2}T\left(z^{\prime }\right)dz^{\prime },$$

where *T*(*z*) is the scattering power. The primed variables in Equation (3) are integration variables (i.e., integration takes place over these variables).

Equation (3) can be derived from the Fermi–Eyges differential equation for the differential fluence.^15^

Equation (3) can be integrated analytically for a square field to obtain:

(5) $$D\left(0,0,z\right)=S_{\rho }\left(z\right)\Phi_{T}\left(0\right)\mathrm{er}f^{2}\left[\frac{X}{4\sqrt{A_{2}\left(z\right)}}\right],$$

where *X* is the side length of the square and erf is the error function.

In polar coordinates ${r^{\prime }}^{2}={x^{\prime }}^{2}+{y^{\prime }}^{2}$ and $dx^{\prime }dy^{\prime }=r^{\prime }dr^{\prime }d\theta$. Inserting this into Equation (3) with *x* = 0 and *y* = 0, and integrating over $r^{\prime }$ results in:

(6) $$D\left(0,0,z\right)=S_{\rho }\left(z\right)\Phi_{T}\left(0\right)\frac{1}{2\pi }\int_{0}^{2\pi }\left[1-e^{-\frac{R^{2}\left(\theta \right)}{4A_{2}}}\right]\;d\theta ,$$

where *R*(*θ*) is the radial distance from the central axis to the aperture boundary. In the case of a circular aperture centered on the beam central axis, Equation (6) can be integrated and set equal to the expression for the dose from a square field (Equation 5) to yield:

(7) $$R^{2}=-4A_{2}\ln\left[1-\mathrm{er}f^{2}\left(\frac{X}{4\sqrt{A_{2}\left(z\right)}}\right)\right].$$

Let us examine the case in which $\frac{X}{4\sqrt{A_{2}\left(z\right)}}\gg 1$. For large values of the argument *w*, the error function has the asymptotic form: $\mathrm{erf}(w)\approx 1-\frac{e^{-w^{2}}}{w\sqrt{\pi }}$.^16^ Substitution of this into Equation (7) shows that, to leading order, X≈2R, independent of the value of *A*_2_(z). The geometric interpretation of this is that the equivalent square is a square that is circumscribed to the circle (or equality of area/perimeter). Now, examine the opposite extreme, in which $\frac{X}{4\sqrt{A_{2}\left(z\right)}}\ll 1$. For small values of *w*, $\mathrm{erf}\left(w\right)\approx \frac{2}{\sqrt{\pi }}w$. In this case, $X\approx \sqrt{\pi }R$, again independent of *A*_2_. The geometric interpretation in this limit is that the area of the square and the circle is equal.

**FIGURE 1:**
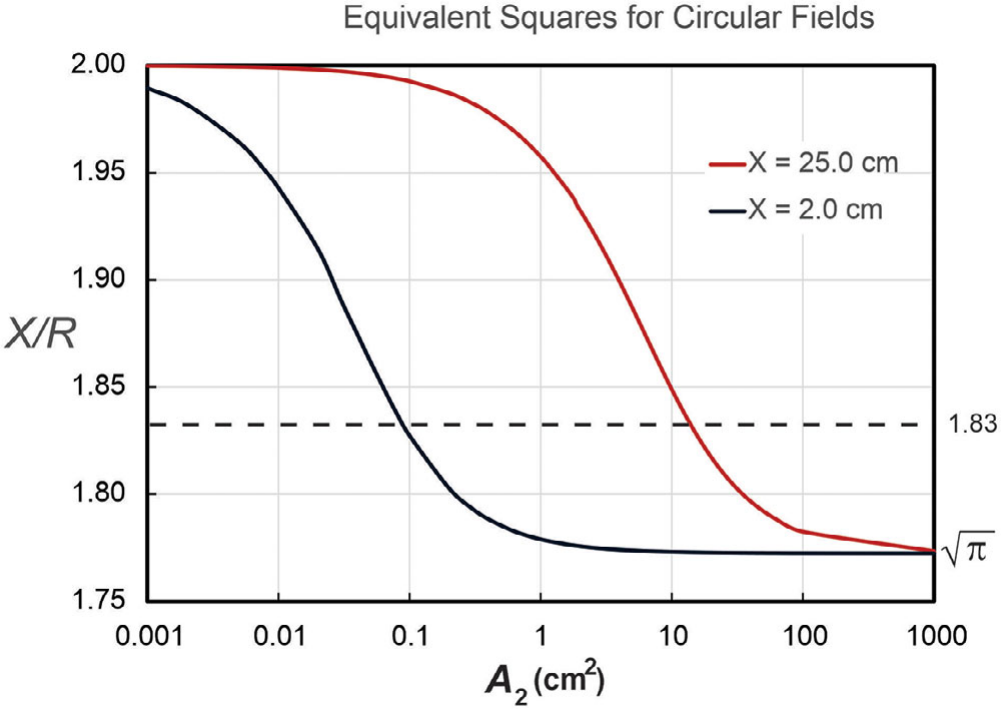
The equivalent square side length *X* divided by the radius of the circle *R* as a function of *A*_2_ from Equation (7) for *X* = 2.0 cm and *X* = 25.0 cm. These are the two extreme bounding values of *X*. The value of *A*_2_ is poorly known, but regardless of the value, the equivalent square ratio must lie between the two curves. That is $\sqrt{\pi }$< *X/R* < 2. *X/R* = 1.83 appears to be the optimum ratio.

The value of *A*_2_(*z*) is not known. This quantity has the units of length squared. It is an energy‐dependent parameter that is a function of depth. Figure 1 shows a plot of *X*/*R* for values of *A*_2_ ranging from 0.001 to 1000 cm^2^ for square fields of side length *X* = 2 and 25 cm. These are the extreme limits of *X*.

The graphs show that for large values of *A*_2_, *X*/*R* = $\sqrt{\pi }$, and that for small values of *A*_2_ that *X*/*R* = 2, as concluded previously. The value of *X*/*R* must lie between these two graphs, and therefore, the value must lie between $\sqrt{\pi }$ and 2, regardless of the beam energy or depth.

To determine the optimum value of *X/R*, tables of output factors were computed for a range of square and circular fields with energies of 6 and 15 MeV and for all applicator sizes. The tables were computed using the RayStation v 2023b (Beam 3D modeling) Monte Carlo algorithm for beam models based on Elekta Versa HD linacs. The grid size was set to 0.2 cm, and the statistical uncertainty of the calculations was 0.3%. The depth of calculation was the nominal value of *d_max_* for each energy. This is the value of *d_max_* for a 10 cm by 10 cm field. In 2022, it was reported that measurements of cutouts for a wide range of energies, applicators, and cutout dimensions agree with RayStation (version 6.0) calculated values within an average difference of “0.5% ± 0.8%.”^17^ In a 2019 report, an extensive collection of cutout measurements was compared to RayStation (version unspecified). The majority of these comparisons were within 3% for SSD = 100 cm, but there were a few outliers for the smallest cutouts; the largest deviation was 5.2% (2 cm by 2 cm, 15 MeV, 10 cm by 10 cm applicator).^18^ The significance of these differences is unknown as no uncertainties are reported.

The RayStation computed tables of output factors for 6 and 15 MeV were used to solve the equation: $S_{e}\left(f_{a},X,100\right)=S_{e}\left(f_{a},R,100\right)$ for values of *R* corresponding to entries for *X*. In that way, a value of *X/R* was computed for each entry in the table of square output values.

The average of the values resulting from the solution of the equation $S_{e}\left(f_{a},X,100\right)=S_{e}\left(f_{a},R,100\right)$ is

(8) X=1.83R.

The value of *X/R* is roughly midway between the two extreme values shown in Figure 1. It remains to test Equation (8) to ascertain its accuracy.

To test the general applicability and accuracy of Equation (8), Monte Carlo dose calculations of the output for 104 square fields with energies of 6, 9, 12, and 15 MeV have been compared to Monte Carlo calculations of the output for the equivalent circular fields given by Equation (8). It is not argued that Monte Carlo calculations should necessarily take precedence over measurements, but there are some difficulties associated with measurements. Cutout evaluation of small electron fields presents some challenges both in fabrication and measurement. Electron cutouts are constructed by hand, and it is somewhat difficult to fabricate a square or circular aperture that is accurately shaped (a perfect square or circle) and is centered on the central beam axis. As an example, a 2 mm error in the size of a square cutout of side length 2.0 cm for 6 MeV results in an output error of about 4%. This would easily mask the agreement described below. The number of cutouts to be constructed would also be prohibitive. A measurement test of the extent described here would require fabrication and measurement of a total of 208 cutouts.

**FIGURE 2:**
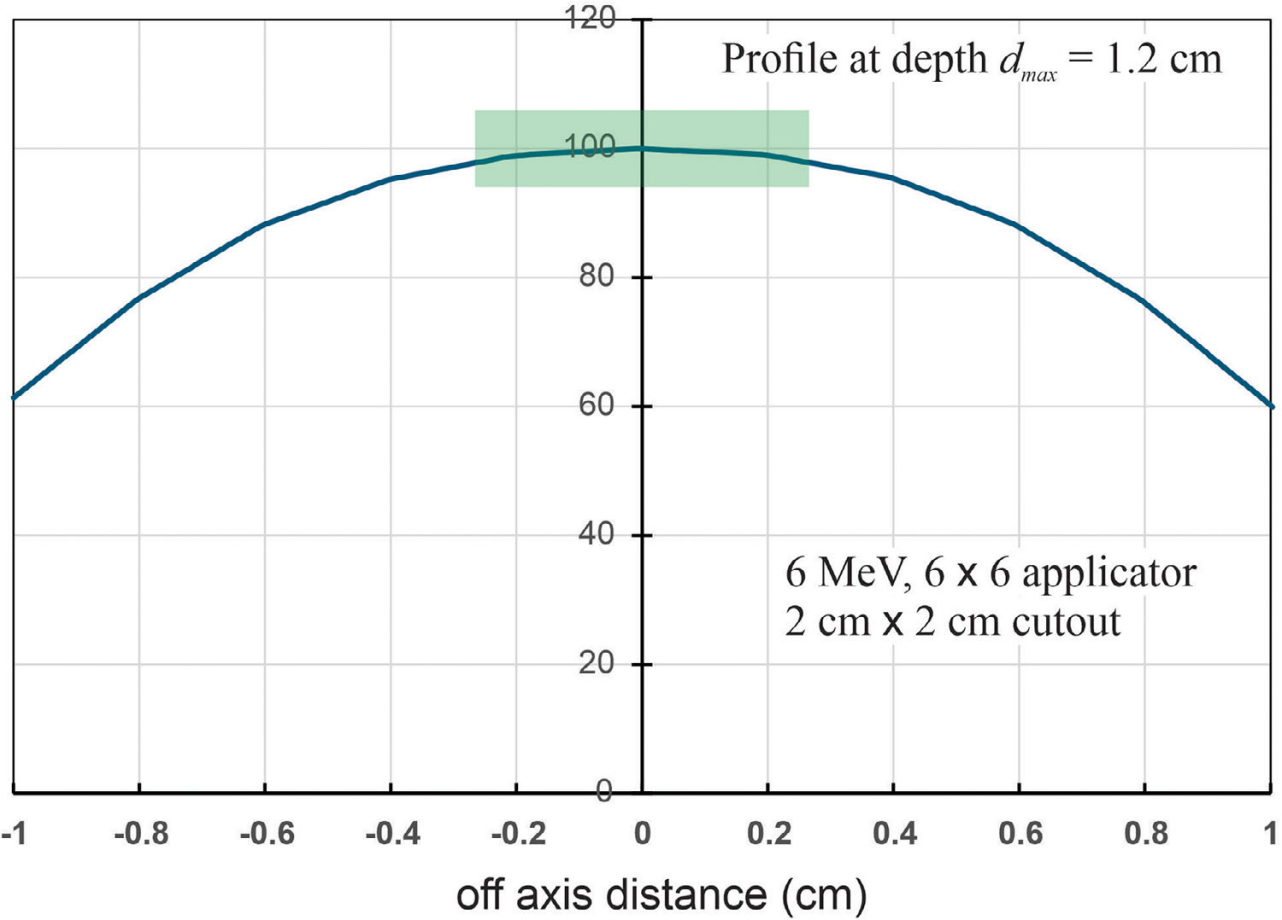
A 6 MeV dose profile for a 2 cm by 2 cm cutout in a 6 cm by 6 cm applicator at a depth of 1.2 cm. The green shaded region shows the extent of a plane parallel ion chamber of diameter 5.0 mm. There will be a small partial volume effect due to the gradient across the ion chamber.

An additional measurement difficulty can be partial volume effects for small fields. It is common to measure output factors using a plane parallel chamber, such as an Advanced Markus chamber. This plane‐parallel chamber has a collecting volume with a diameter of 5 mm. For a 2.0 cm by 2.0 cm cutout, there is a small gradient in the dose across the diameter of the collecting volume. Figure 2 shows the axisymmetric profile of the dose distribution perpendicular to the central axis for a 2 cm by 2 cm 6 MeV beam at a depth of 1.2 cm (nominal *d_max_* for a 6 MeV beam). The reading of the plane parallel chamber will reflect the average dose over this profile and will thus be lower than the dose on the central axis. The dose on the central axis is $D_{0}=\bar{D}\times \mathrm{CF}$, where $\bar{D}$ is the measured dose, and CF is a correction factor. An estimate of the CF is:

(9) $$CF^{-1}=\frac{2}{R^{2}}\int_{0}^{R}r\;f(r)dr$$

where *f* (*r*) is the normalized beam profile (*f* (0) = 1.00). Equation (9) is derived by calculating the average of the dose over the cylindrical collecting volume. For the *R* = 2 cm case (6 MeV), the correction factor is about 1.3%. Failure to account for this in the measurements will result in a systematic error in comparing output factors to *calculated* values. If, however, square field measurements are compared to circular field measurements, this systematic error is likely to roughly cancel. Adding to the challenges described above, many published measurement comparisons do not report measurement uncertainties. This makes it difficult to assess the significance of discrepancies—the reported differences may not be real.

The Monte Carlo calculations have been performed in a water phantom using the RayStation software (2023b) with a statistical accuracy of 0.3% and a grid size of 2 mm. The electron energies are 6, 9, 12, and 15 MeV using Elekta applicators of size 6 × 6, 10 × 10, 14 × 14, 20 × 20, and 25 × 25 cm^2^ (defined at SSD = 95 cm). The results are not expected to be any different for Varian linacs to the extent that the electron beam characteristics are the same. Square fields were calculated first with side lengths ranging from 2.0 cm to 10.0 cm, depending on the applicator. The corresponding circular field outputs were then computed for comparison.

In RayStation, it is possible to very accurately define small square and circular apertures that are precisely centered on the beam central axis, and the point dose can be evaluated at the precise depth of nominal *d_max_* on the central axis in liquid water.

Square field sizes larger than 10 cm are expected to be in lateral side‐scatter electron equilibrium: ($R>0.88\sqrt{E_{p,0}}$), where *E_p_*_,0_ is the most probable energy at the surface in MeV. Square field sizes smaller than 2.0 cm are not relevant. The reason is that the distance from the 50% line to the 90% line for electron fields is about 1 cm, and therefore, the margin around every target should be about 1 cm on each side. For small fields, the percent depth dose (PDD) curve can shift significantly toward the surface due to a lack of lateral electron equilibrium. Equation (8) was derived at the depth of the nominal *d_m_*. This is the value of *d_m_* for large fields. This equation will provide the output at the same fixed depth for both the circular and the square field, *even though that depth may no longer be the actual depth where the dose is maximum*. If the dose is prescribed to a depth of (actual) *d_m_*, *d*_80_, or *d*_90_ for that field size, a depth dose correction may be needed to convert the dose from the nominal *d_m_* to the specified depth.

The shift in the depth dose is expected to be most pronounced for the highest energy. Monte Carlo calculations have been performed to evaluate the circumstances under which the PDD at the nominal *d_m_* departs significantly from 100%. These results are relatively independent of applicator size.

## 3 RESULTS

**FIGURE 3:**
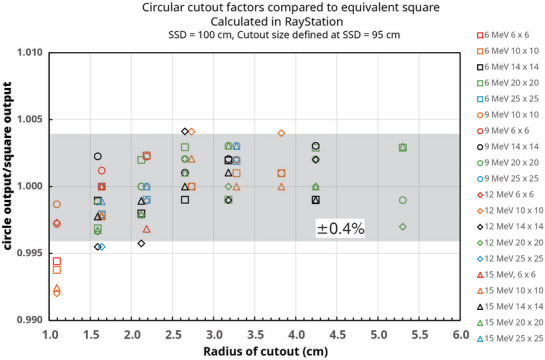
The ratio of the output for circular fields to the output of the predicted equivalent square fields, both computed by the Monte Carlo algorithm, as a function of the radius of the circular field. The size of the predicted equivalent square fields was computed from Equation (8). The dimensions listed in the legend are for the applicator size. The shaded band represents the expected ±0.4% uncertainty in the ratio (1σ). The equivalent square rule (Equation 8) is accurate to better than 1%.

Figure 3 shows the ratio of the computed output for circular apertures to the computed output for the equivalent square field based on Equation (8). The uncertainty in the RayStation calculations is 0.3% and therefore the uncertainty in the ratio is 0.4%. This is represented by the shaded band in Figure 3. Accuracy is worst for small cutouts and low energy. Most of the data point ratios shown in Figure 3 are within 0.5% of 1.000, and all are within 1.0%. The average ratio over all 104 combinations of energies, applicators, and apertures is 1.000 with a standard deviation of 0.003.

McParland has considered equivalent fields for electron beam central axis dose calculations.^10^ This author reports *X* = 1.72 *R*. A formula for the equivalent square of circular fields has been derived previously by Birgani et al.^19^ This formula has an energy dependence: *X*(cm) = 2 *R*− (0.0134 *E*(MeV) + 0.13), where *E* is the energy in units of MeV. This formula generally predicts values of *X* that are lower than predicted by Equation (8) by up to 8%.

**FIGURE 4:**
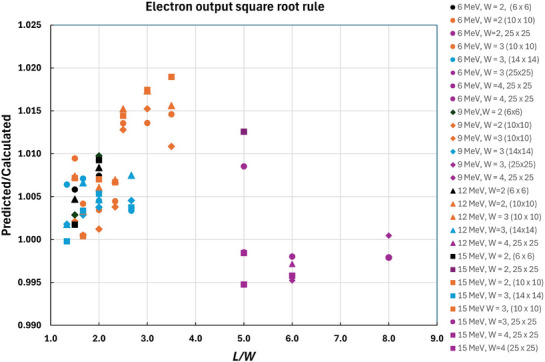
The ratio of the predicted output from the square root rule to the output for the rectangle, both calculated by the Monte Carlo algorithm, as a function of the length over the width. The square root rule is accurate to better than 2%.

The accuracy of Equation (8) has been compared with that of the square root rule (Equation 2). The output for rectangular apertures has been computed using the Monte Carlo algorithm and compared to the prediction of the square root rule using Monte Carlo calculated square field outputs. Figure 4 shows the ratio of the predicted output to the Monte Carlo computed output as a function of *L/W*. The uncertainty in the ratios shown in Figure 4 is 0.4% (1 σ). The square root rule tends to slightly over‐estimate the output for applicators of 14 × 14 cm^2^ and smaller. For all the parameters examined, the square root rule accurately predicts the output to within 2%. The rule for equivalent squares of circles (Equation 8) appears to be more accurate than the square root rule for the parameters considered.

**FIGURE 5:**
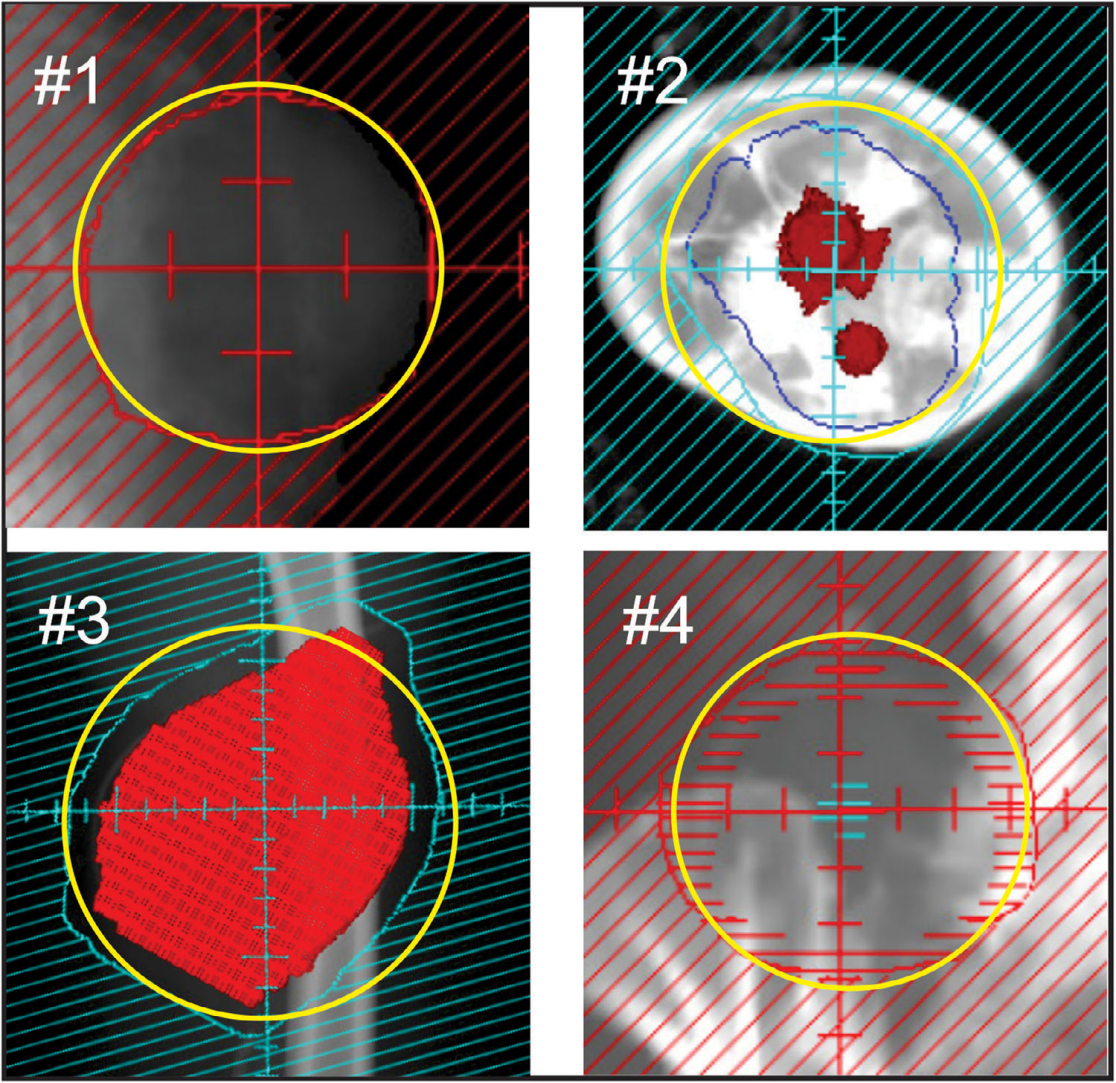
The four clinical apertures listed in Table 1. The diagonal lines show the blocked regions. The scale for each aperture is different. The tick marks are 1.0 cm apart. The yellow circles represent the approximate equivalent circle drawn by eye before any dose estimates were calculated. The horizontal red dash marks for #4 represent a target volume, not the edge of the aperture. Table 1 shows that the estimated output values agree with measurements to within a few percent.

A number of clinical examples of the application of Equation (8) are displayed in Table 1 and in Figure 5. The figure shows the apertures corresponding to each ID number in the table. The purpose of the table and the figure is to show how Equation (8) can be applied in the clinic and the accuracy that can be expected. These examples were chosen from our clinic for inclusion in this report *without knowing the level of accuracy beforehand* (these examples were not “cherry picked”). Superimposed on the apertures in Figure 5 are the estimated circular apertures. Table 1 shows the estimated value of *R* and the corresponding value of *X*. The estimated output is found by interpolating in a table of measured output values for square fields compiled at the time of commissioning of these Elekta Versa linacs. The estimated output values are compared to the measured values for these patients.

**TABLE 1:** Clinical examples.

ID #	Energy (MeV)	Applicator size (cm)	Patient diagnosis/site	Estimate of *R* (cm)	Equivalent square *X* (cm)	Estimated output (cGy/MU)^a^	Measured output (cGy/MU)^b^	Estimated/Measured
1	6	6×6	Squamous cell, skin	2.0	3.7	0.846	0.836	0.988
2	9	20×20	Squamous cell, scalp	6.0	11.0	0.987	0.970	1.018
3	6	25×25	T‐cell lymphoma, thigh	6.5	11.9	1.031	1.010	1.021
4	9	10 x 10	Ovarian ca, pelvis	3.0	5.5	0.975	0.980	0.995

^a)^ At the depth of nominal *d_max_*, SSD = 100 cm.

^b)^ Measurements were made with an Advanced Markus chamber in a solid water phantom at the charted *d_max_* depth.

It can be seen from Table 1 that the equivalent square formula (8) is reasonably accurate in predicting the measured output. It can be concluded that Equation (8) is a viable equivalent square formulation that can be used to make quick output estimates for checking treatment plans. If there is any doubt, one should always make a measurement, keeping in mind the caveats discussed in Section 2.

**FIGURE 6:**
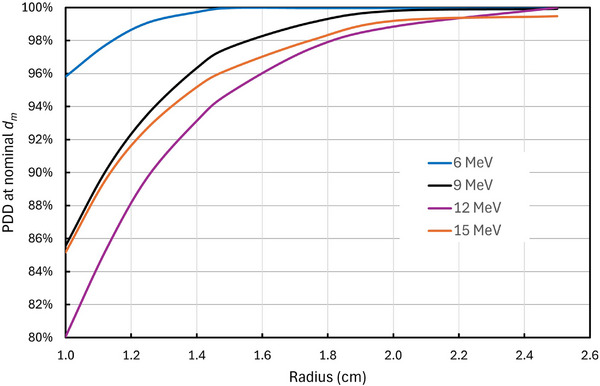
The percent depth dose at the nominal depth of maximum dose (defined as *d_m_* for a 10 cm by 10 cm field size) as a function of the radius for circular apertures. For small apertures, the depth dose shifts toward the surface and therefore the PDD at the nominal *d_m_* decreases. For *R* > 1.8 cm, the decrease from 100% is less than 2%.

As described in Section 2 (Methods), the effect of the shift of the PDD curve has been evaluated for a small field size. Figure 6 shows the value of the PDD for small circular fields at the depth of the nominal *d_m_*. The nominal *d_m_* is the depth of maximum dose for a 10 cm by 10 cm field aperture. As the field size decreases, the PDD curve shifts toward the surface, and the PDD at the depth of the nominal *d_m_* decreases. These results were calculated using the RayStation Monte Carlo software and are relatively independent of applicator size. Figure 6 shows that the PDD does not deviate from 100% by more than 2%, provided that *R* > 1.8 cm, for beam energies of 6–15 MeV. For 6 MeV, the PDD at nominal *d_m_* is greater than 98% for *R* > 1.2 cm. Thus, it can be concluded that the output at the actual *d_m_* for the given aperture will not differ significantly from the output at the nominal *d_m_* for the conditions stated above.

## 4 CONCLUSION

Many electron apertures are more circular than rectangular. A very simple formula for computing the equivalent square of circular electron fields has been presented, viz, *X* = 1.83 *R*. This formula has been shown to predict circular aperture output with an accuracy of better than 1% for a wide range of field sizes, energies, and applicators. For rectangular fields, the square root formula should be used. For apertures that are more circular than rectangular, the formula presented here can be used with a high degree of confidence. If there is any doubt, a measurement should be made.

An estimate of circular or approximately circular cutout output can be obtained by constructing a best‐fit circle and using Equation (8) to find the equivalent square. Interpolation of output values in a table of square cutouts for the specific energy and applicator size will then provide an estimate of the output for the field.

## AUTHOR CONTRIBUTIONS

This is a single‐author manuscript.

## CONFLICT OF INTEREST STATEMENT

The author declares no conflicts of interest.
